# PTEN-regulated PI3K-p110 and AKT isoform plasticity controls metastatic prostate cancer progression

**DOI:** 10.1038/s41388-023-02875-4

**Published:** 2023-10-24

**Authors:** Karina A. Miller, Seamus Degan, Yanqing Wang, Joseph Cohen, Sheng Yu Ku, David W. Goodrich, Irwin H. Gelman

**Affiliations:** 1 Department of Cancer Genetics & Genomics, Roswell Park Comprehensive Cancer Center, Elm and Carlton Streets, Buffalo, NY 14209 USA; 2 Department of Pharmacology & Therapeutics, Roswell Park Comprehensive Cancer Center, Elm and Carlton Streets, Buffalo, NY 14209 USA; 3 Dana Farber Cancer Institute and Harvard Medical School, Boston, MA USA; 4 Present Address: American Society of Human Genetics, Rockville, MD 20852 USA; 5 Present Address: Sequence, Inc., Morrisville, NC USA

**Keywords:** Prostate cancer, Cancer genomics

## Abstract

*PTEN* loss, one of the most frequent mutations in prostate cancer (PC), is presumed to drive disease progression through AKT activation. However, two transgenic PC models with Akt activation plus *Rb* loss exhibited different metastatic development: *Pten/Rb*^*PE:−/−*^ mice produced systemic metastatic adenocarcinomas with high AKT2 activation, whereas *Rb*^*PE:−/−*^ mice deficient for the Src-scaffolding protein, Akap12, induced high-grade prostatic intraepithelial neoplasias and indolent lymph node dissemination, correlating with upregulated phosphotyrosyl PI3K-p85α. Using PC cells isogenic for PTEN, we show that PTEN-deficiency correlated with dependence on both p110β and AKT2 for in vitro and in vivo parameters of metastatic growth or motility, and with downregulation of SMAD4, a known PC metastasis suppressor. In contrast, PTEN expression, which dampened these oncogenic behaviors, correlated with greater dependence on p110α plus AKT1. Our data suggest that metastatic PC aggressiveness is controlled by specific PI3K/AKT isoform combinations influenced by divergent Src activation or PTEN-loss pathways.

## Introduction

Prostate cancer (PC) remains the most commonly diagnosed non-cutaneous cancer and the second highest contributor to cancer-related deaths in U.S. men [1]. While localized PC has a near 100% survival rate, patients with presumed localized disease can have disease recurrence years after the removal of the prostate, suggesting that dissemination of PC cells occurs early in tumor progression [2, 3]. Approximately 20–30% of PC patients progress to biochemical recurrence and metastatic disease (mPC) within 5 years of radical prostatectomy [4, 5]. Furthermore, disseminated tumor cells (DTCs) can be detected in the bone marrow of prostate cancer patients with clinically localized disease [6], further suggesting that the spread and colonization of distant sites can occur early during tumor development.

AKAP12 (also known as SSeCKS, Gravin, or AKAP250) functions as a metastasis suppressor in prostate cancer models [7–9] based on its ability to scaffold kinases such as Src, PKC, and PKA, and to suppress their activation of downstream signaling mediators [10]. AKAP12’s ability to mediate temporospatial signaling control is facilitated through its membrane binding domains, including an N-terminal myristylation signal and three polybasic phosphoinositol phosphate-binding domains [11], and an F-actin binding domain [12]. AKAP12 scaffolds Src away from integrin-FAK-growth factor receptor membrane complexes to caveolin-rich lipid rafts. Although AKAP12 binding does not suppress intrinsic Src tyrosine kinase activity it suppresses Src-mediated MEK-ERK1/2, STAT3, and PI3K/AKT signaling that controls oncogenic progression parameters such as tumor invasiveness, anchorage-independent growth, and survival [8, 13]. Approximately one-third of human PC metastases show chromosomal loss of 6q24–25.2, which encodes the *AKAP12* locus [14], and additionally, a significant number of PC metastases exhibit the transcriptional downregulation of *AKAP12* [10] due to promoter hypermethylation [15]. This parallels clear evidence showing increased activation of Src-family kinases (SFK) in PC progression, as monitored by a shared poY416 auto-phosphorylation site [16] or phosphoproteome signatures [17]. The loss of Akap12 in mouse embryo fibroblasts induced an Rb-dependent premature senescence associated with multinucleation [18], consistent with findings of increased senescence markers, hyperplasia, and relative Akt^poS473^ levels in the prostate epithelial cells of *Akap12*^*−/−*^ mice [19]. Indeed, *Akap12*^*−/−*^*;* *Rb*^*PE:−/−*^ transgenic male mice in the C57BL/6 background developed high-grade prostatic intraepithelial neoplasias (HG-PIN), and consistent with AKAP12’s role as a metastasis-suppressor [10], these mice exhibited dissemination to local lymph nodes of indolent, transitional PC cells that express both basal and luminal epithelial markers [3].

Dysregulation of the PI3K/AKT signaling pathway plays an essential role in the survival, proliferation, metabolism, and motility in cancer progression [20] in both primary and metastatic PC [21–23]. In rare cases, PI3K/AKT activation involves either (i) point mutations in *PIK3CA*, resulting in increased activity of the PI3K-p110α, (ii) amplification of *PIK3CA* or *PIK3CB*, the latter encoding the PI3K p110β isoform, or (iii) kinase-activating point mutations in AKT1 [24, 25]. In contrast, PTEN loss (due to deletion, mutation, and/or downregulation), is one of the most frequent mutations in primary PC, and as well, a strong predictor of poor biochemical recurrence and recurrence-free survival [26–28]. As a critical inhibitor of the PI3K/AKT pathway, PTEN loss correlates with activated AKT, as determined by relative levels of phosphoSer473 (poS473) (Nomenclature as per Aasland et al. [29]) and phosphoThr308 (poT308)-AKT [30, 31]. However, a growing body of literature strongly suggests that PC progression is likely controlled by subtle interplays between p110 and AKT isoforms [32]. For example, PI3K activation in PTEN-deficient PC cells was shown to be receptor tyrosine kinase-independent, yet dependent on p110β and p110δ, whereas activation of HER2 by heregulin was p110α-dependent [33]. Schwartz et al. [34] showed that treatment of PTEN-deficient LNCaP cells with a p110β-specific inhibitor induced survival through a compensatory activation of p110α and HER3. In contrast, a transgenic (Tg) mouse model in which PC is driven by the prostate-specific expression of polyoma middle T antigen, a known binder and activator of Src [35], is much more dependent on p110α [36]. Zhang et al. [37] clarified that the p110β preference in PTEN-deficient cells correlates with increased binding of CRKL to PI3K-p110β, and that the increased binding of CRKL to tyrosine phosphorylated p130Cas in these cells is responsible for their increased co-sensitivity to both PI3K-p110β and Src inhibitors. Lastly, several studies recently showed that the survival of PTEN-deficient PC cells in anchorage-independent conditions relied more on AKT2 than on AKT1 [38, 39].

By comparing Tg models, we sought to determine whether Akt activation was sufficient to induce primary and metastatic PC when combined with another known progression mutation, *Rb* deletion. Interestingly, *Pten;Rb*^*PE:−/−*^ and *Akap12*^*−/−*^*;* *Rb*^*PE:−/−*^ mice express similarly increased levels of activated Akt, based on relative Akt^poSer473^, in their prostates. However, whereas the *Pten/Rb-*null model progressed to adenocarcinoma and systemic, aggressive metastasis formation [40], the *Akap12/Rb-*null model produced HG-PIN with the dissemination of indolent metastatic cells to local lymph nodes [3]. These data indicate that Akt activation alone cannot explain the different pathologies, and thus, we addressed whether they were controlled by differing dependencies on p110 and Akt isoforms, and whether PTEN status affected these dependencies. To address this, we produced isogenic pairs of human PC lines as well as *Pten/Rb-*null Tg PC lines that differed only in their PTEN expression. Our data indicate that multiple parameters of survival, metastatic growth, and invasiveness of PTEN-deficient PC cells were more dependent on AKT2, and in some cases, on AKT3, whereas the re-expression of PTEN, which dampened oncogenic behavior, converted dependency to AKT1. Moreover, inhibition of clonogenic survival and chemotaxis of PTEN-deficient PC cells required the combined use of p110β plus AKT2 inhibitors, whereas for PTEN-positive cells, p110α plus AKT1 inhibitors had the greater effect. Our data strongly suggest that the clinical targeting of the PI3K-AKT axis in PC requires knowledge of PTEN status followed by the inhibition of the appropriate p110/AKT isoforms.

## Materials and methods

### Cell culture

22Rv1 and PC3 were obtained from ATCC (Manassas, VA). LNCaP depleted of fibroblasts was a generous gift from Shahriar Koochekpour (Univ. of Florida), and LNCaP-C4-2B[luc-GFP] was a gift from Dean Tang (Roswell Park Comprehensive Cancer Center [RPCCC]). The T402 cell line isolated from Pb-Cre:*Pten*^fl/fl^:*Rb1*^fl/fl^ C57BL/6 mice, was kindly provided by David Goodrich (RPCCC). All cell lines were maintained at 37 °C/5% CO_2_ humidified incubator. 22Rv1 were grown in DMEM (Corning, Corning, NY) supplemented with 10% fetal bovine serum (FBS; ThermoFisher, Grand Island, NY) and 1x penicillin/streptomycin (P/S; Corning). LNCaP and LNCaP-C4-2B[luc-GFP] were cultured in RPMI-1640 media (Corning) supplemented with 10% FBS and 1x P/S, with the latter grown with puromycin (2 μg/mL). T402 was maintained in Prostate Epithelial Media (PrE) [40]. MAT-LyLu (MLL) rat prostate cancer cells with Tet^OFF^-regulated SSeCKS/Akap12 expression [9] were grown in DMEM/10% FBS supplemented with 8 μg/ml of hygromycin, 1 μg/ml puromycin and 0.7 μg/ml tetracycline. All cell lines were authenticated using STR profiling and tested by PCR for the lack of mycoplasma (Roswell Park Genomics Shared Resource).

### sh/siRNA

The RPCCC Gene Modulation Core provided the PTEN shRNA clones (Dharmacon) V2LHS_192536, V2LHS_92314, and control pGIPZ. Cells were infected with high-titer lentivirus containing the shRNA sequences and selected using puromycin (2 µg/mL). si/shRNA sequences are described in Supplementary Table S2.

### siRNA transfection

Cells at 50–80% confluence in 6-well tissue culture plates were transfected with siRNA in Lipofectamine 2000 or 3000 (Invitrogen) as per manufacturer’s protocol, and then cell lysates were analyzed after 48–72 h.

### Plasmids

PTEN-GFP plasmid (#30391; Addgene, Watertown, MA) was validated by sequencing (primer 5′-CCAAGGACCTGAAATGACCC-3′) and by immunoblotting (IB) (87 kDa). pBABE-puro-AKT1^S473D^ and -AKT2^S474D^ [41] were generous gifts of David Guertin, Univ. Mass. Med. School.

### Inhibitors

Inhibitors were obtained from MedChem Express (Monmouth Junction, NJ) and diluted in DMSO. Inhibitors used: AKT2 (CCT-128930, #HY-13260), AKT1 (A-674563, #HY-13254), p110β (GSK-2636771, #HY-15245), p110α (BYL-719, #HY-15244), Src (Saracatinib, #HY-10234).

### Invasion and migration assays

Chemotaxis and invasion assays were performed using 24-well transwell inserts with 8.0 µm pore PET membranes (Falcon, Corning, NY) as described previously [8], seeded in triplicate with 5 × 10^4^ (T402) or 1 × 10^5^ (LNCaP) cells in the upper chamber containing 0.5% FBS media, and 10% FBS media in the lower chamber. Inhibitor concentrations: A-674563 (AKT1i) 100 nM, CCT-128930 (AKT2i) 300 nM, BYL-719 (p110αi) 5 nM, GSK-2636771 (p110βi) 25 nM, Saracatinib (Srci) 150 nM. The cells were incubated in a 37 °C humidified incubator for 16–24 h for chemotaxis assays, and 24–48 h for invasion assays. Inserts were stained using Fisher’s PROTOCOL Hema3 stain kit (Hampton, NH) and cells counted using ImageJ software.

### Proliferation assays

Transfected or treated cells (1-2 × 10^3^) were seeded on 96-well plates and allowed to grow for the indicated times. Cells were washed with PBS, fixed with ice-cold 100% methanol for 10 min at −20 °C, and stained with 0.5% crystal violet. Dried plates were solubilized with 10% acetic acid for 20 min at room temperature, and absorbance read at 595 nm.

### Methylcellulose and anoikis assays

Cells were grown in 3D by suspending in media containing 1.3% methylcellulose (Sigma, St. Louis, MO) on plates pre-coated with 0.4% agarose, or grown in suspension atop 1.4% agarose-coated plates with complete (+10% FBS) or serum-depletion (+0.5% FBS) media. CellTiter-Glo 3D Cell Viability Assay (#G9681; Promega, Madison, WI) was used to determine the number of viable cells in 3D cell culture according to the manufacturer’s protocol. An opaque-walled 96-well plate (Corning) compatible with the Veritas Microplate Luminometer (Turner BioSystems, Sunnyvale, CA) was used, and the luminescence signal recorded according to the manufacturer’s protocol.

### Clonogenic assay

Survival of adherent cells was performed as described [42], using 200 cells/well for T402 and 22Rv1, and 400 cells/well for LNCaP in 24-well plates.

### RNA-Seq

Prostates were harvested from *Akap12*^−/−^;Pb-Cre:*Rb1*^fl/fl^ and Pb-Cre:*Pten*^fl/fl^:*Rb1*^fl/fl^ mice by microdissection, snap frozen in liquid nitrogen, and stored at −80 °C. RNA was isolated from frozen tissue using TRIzol (Invitrogen) according to the manufacturer’s instructions. RNA samples were interrogated by the RPCCC Genomics Shared Resource using TruSeq Stranded Total RNA with the RiboZero Gold library prep kit (Illumina, San Diego, CA) with 1 μg input, sequenced on an Illumina HiSeq2500. Alignment to the mouse genome (mm10 version) was performed by the RPCCC Bioinformatics Shared Resource using RefSeq [43] and the UCSC Genome Browser [44]. Quality control for the raw reads was performed using *fastqc* [45] and adapter trimming was done using *atropos* [46]. Spliced alignments of reads to the reference genome was done using TopHat2 [47], allowing a maximum of 1 mismatch/read, and quality control for this alignment was done using RSeQC software [48]. The differential expression report was generated using DESeq2 [49] and the expression levels were normalized using Fragments Per Kilobase of transcript per Million mapped reads (FPKM). FASTQ files for the RNA seq data were uploaded to the Gene Expression Omnibus (GEO) under accession numbers GSE90891 and GSE242387.

### Immunoblotting (IB)

Tissue was homogenized, or cultured cells washed, and then lysed in complete RIPA buffer as described previously [50]. Primary antibodies (Ab) used: AR (sc-816), PI3K p110β (sc-376641), β-actin (sc-47778), GAPDH (sc-32233), and α-tubulin (sc-5286) from Santa Cruz Biotechnology (Santa Cruz, CA); AKT1 (#2938), AKT2 (#3063), AKT3 (#8018), AKT3 (#14982), AKT^poS473^ (#9018), AKT^poS473/4^ (#9271), AKT2^poS474^ (#8599), AKT^poT308^ (#4056S), phospho-AKT substrate RXXS*/T* (#9614S), PRAS40 (#2691S), PRAS40^poT46^ (#2997T), PTEN (#9552), and PI3K p110α (#4249) from Cell Signaling Technologies (Beverly, MA); SMAD4 (ab-40759) from Abcam (Cambridge, MA); SSeCKS/Akap12 [12]. Between 15 and 35 µg of total protein per sample was separated by SDS-PAGE for IB.

### Immunoprecipitation (IP)

IP was performed as described [51] using protein A/G-PLUS-Agarose beads (Santa Cruz) and 1:50 dilutions of Abs for AKT1, AKT2 or AKT3 (above), followed by IB using the same isoform Abs or Ab for AKT^poS473^ (above), or p85α Ab (#4292, Cell Signaling), followed by IB using anti-p85α or anti-phosphotyrosine Ab (4G10, #96215, Cell Signaling).

### Immunohistochemistry (IHC)

IHC was performed as described [9] using AKT^poSer473^ Ab (#4060, Cell Signaling) at 1:100. Images were scanned using the Aperio Digital Pathology Slide Scanner (Leica Biosystems, Buffalo Grove, IL).

### Animal experiments

All animal experiments were performed with the approval of the Roswell Park Institutional Animal Care and Use Committee. The *Akap12*^−/−^;Pb-Cre:*Rb1*^fl/fl^ (*Akap12*^−/−^;*Rb1*^*PE:−/−*^, “*Akap12/Rb-*null”) HG-PIN mouse model was developed and published previously [3]. The Pb-Cre:*Pten*^fl/fl^:*Rb1*^fl/fl^ (*Pten*;*Rb1*^*PE:−/−*^, “*Pten/Rb-*null”) mouse was developed in D. Goodrich lab (RPCCC) and previously published [40]. Both models are in the C57BL/6J background. Prostates and pelvic lymph nodes were collected from euthanized 10–18 month-old males. Orthotopic tumors were produced by injecting 200,000 T402-Luc and T402[PTEN]-Luc cells suspended in 50 μL of Matrigel into the anterior and dorsal lobes of male SCID mice and allowed to grow until tumors reached roughly 250 mm^3^ by IVIS (below). Mice were treated (6 mice/group) for 5 weeks with daily IP of vehicle (50% DMSO, 40% PEG-300, 10% Tween-80), p110αi (20 mg/kg), p110βi (30 mg/kg), AKT1i (25 mg/kg), AKT2i (30 mg/kg), p110α/AKT1i, or p110β/AKT2i, and weight checked daily. Tumor measurements were done via IVIS imaging (Spectrum, Perkin-Elmer) every 3 days. In vivo metastasis assays were performed according to the technique of Havens et al. [52]: 2 × 10^5^ LNCaP-C4-2B[luc-GFP] cells were injected subcutaneously in 100 μL Matrigel into male SCID mice. The next day, mice were treated daily by IP for 3 weeks with vehicle, p110βi (30 mg/kg), AKT2i (30 mg/kg), or the p110βi/AKT2i combination. Livers and kidneys removed from euthanized mice were snap-frozen and stored at −80 °C until DNA extraction, whereupon DNA was isolated from tissue thawed on ice using Qiagen DNeasy Blood and Tissue kits according to the manufacturer’s specifications. Quantitative PCR reactions were run using 10 µL of 2x PowerUp SYBR Green Master Mix, 100 nM of either mouse β-actin forward and reverse primers or human *Alu* forward and reverse primers, 200 ng of DNA, and water up to 20 µL per reaction (Supplementary Table S2). The thermal conditions in an Applied Biosystems Veriti were 95 °C for 10 min followed by 40 cycles of 95 °C for 15 s and 60 °C for 1 min. Relative human *Alu* gene levels were normalized to mouse actin gene levels. The statistical power for tumor or metastasis formation was obtained by Student’s *t* test (2-sided α = 0.05, *n* = 6 or 5 per group, coefficient of variation = 0.6). Sample sizes used established power of >80%. No randomization was used between groups, and tumor/metastasis formation data were calculated for blinded groups, which were then unblinded by the investigators.

### Data analysis and statistics

The majority of data analysis and figure generation was performed in GraphPad Prism v7. Significance in the Oncomine database (ThermoFisher) was defined as non-overlap of first quartile primary prostate tumor samples and third quartile of metastatic prostate samples. Oncoprints and survival curves were generated from cBioPortal [53] for primary and mPC samples. Error bars reflect s.d. using Student’s *t* test, and statistical significance is reported as *p*-values of under 0.05. Variance between groups or replicates in a given experiment were <15%.

## Results

### AKT activation is not sufficient to induce prostatic adenocarcinoma

**Fig. 1:**
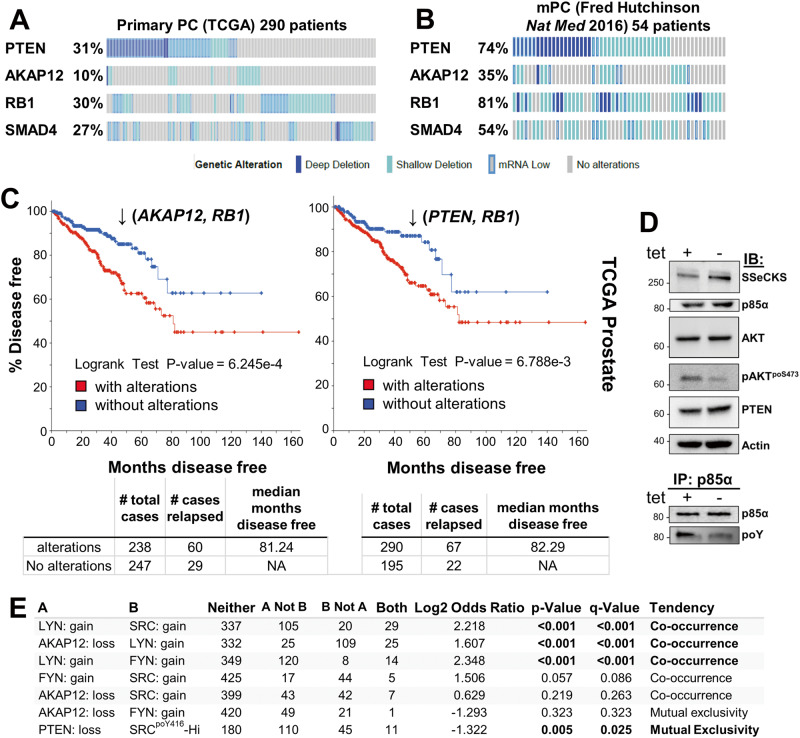
Loss of *PTEN, AKAP12, RB1*, and *SMAD4* in human primary and metastatic prostate cancer datasets. cBioPortal oncoprints comparing *PTEN*, *AKAP12*, *RB1* or *SMAD4* loss (TCGA Prostate Firehose vs. Fred Hutchinson CRC: hetloss, homdel, exp < −2) in primary (**A**) vs. metastatic PC (**B**). **C** Disease-free progression analysis of TCGA prostate (Firehose) samples for the combined loss of *AKAP12* plus *RB1*, or *PTEN* plus *RB1* (top panel), as well as number of total cases, number relapsed, and median number of months disease free (bottom panel). **D** *Upper panel*. IB analysis of MATLyLu PC cells with Tet^Off^-regulated SSeCKS/Akap12 re-expression, grown overnight in media with or without 0.7 μg/mL tetracycline (“tet + or −”). *Lower panel*. IP of p85α followed by IB for either p85α or total phosphotyrosine (poY). **E** Co-occurrence analysis generated by cBioPortal TCGA Prostate Firehose (492 samples) of *LYN*, *FYN* or *SRC* gain (gain, amp, exp >2) with *AKAP12* loss (hetloss, homdel).

The loss of *PTEN* is one of the most prevalent changes in primary PC disease [54] (Fig. 1A). PTEN loss is a likely marker of cancer initiation because biallelic deletion of *Pten* in transgenic mouse models is sufficient to induce HG-PIN in less oncogenic backgrounds such as C57BL/6 [55]. The loss of PTEN lipid phosphatase activity is thought to activate oncogenic AKT by allowing it to bind through its intrinsic PH domain to PI3K-generated PIP3 at the plasma membrane [56, 57]. Indeed, *PTEN* loss correlates with increased AKT activation levels in more advanced cases of human PC, based on increased relative levels of AKT^poS473^ staining [58, 59], serving as a strong predictor of biochemical recurrence [60]. Progression to prostatic adenocarcinoma and distal metastases requires additional losses in tumor suppressors such as *Rb* or *Smad4* [40, 61], mimicking their frequent losses in primary PC (Fig. 1A). The notion that *PTEN, RB1*, or *SMAD4* play important roles in regulating metastatic PC (mPC) is evidenced by gene losses that are more frequent in mPC than in primary lesions (Fig. 1A, B; Supplementary Fig. S1A). Consistent with AKAP12’s known metastasis suppressor function [10], *AKAP12* loss is 3.5-fold more frequent in mPC than in primary PC (Fig. 1A, B).

**Fig. 2:**
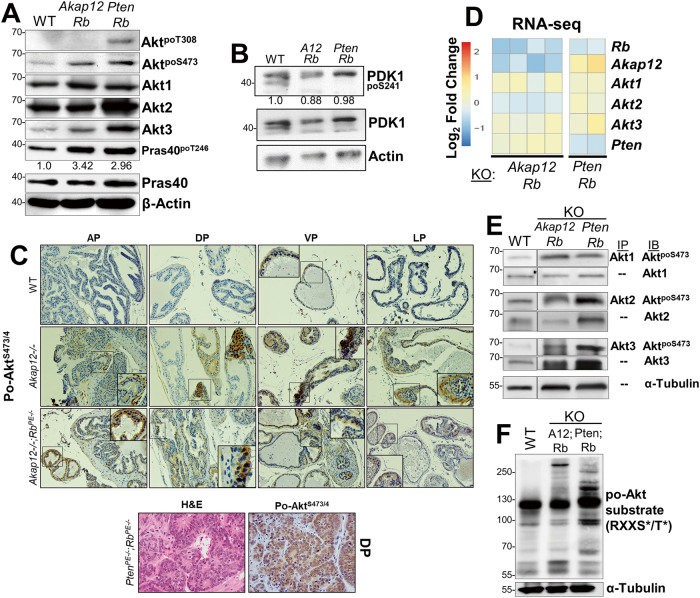
AKT isoform differences in transgenic PC mouse models. **A** IB analysis of prostate lysates from WT, *Akap12/Rb-* or *Pten/Rb-*null male mice for Akt isoforms, total AKT poS473 or poT308 levels, total vs. poT248 levels of the pan-AKT substrate, Pras40, with β-actin serving as loading controls. Fold differences in relative Pras40^poT248^ levels are shown. **B** Lysates from panel A probed for total PDK1, PDK1^poS241^, or β-actin. **C** *Top-* IHC staining for Akt^poS473/4^ in paraffin-embedded microdissected prostate lobes (AP, anterior; DP, dorsal; VP, ventral; LP, lateral) at 200X (main image) or 400X (inset) magnification. *Bottom-* IHC staining (H&E or AKT^poS473^ in DP sections from *Pten/Rb-*null mice. **D** Heat map comparison from RNA-seq analysis of *Rb, Akap12, Akt1, Akt2, Akt3* and *Pten* RNA levels in *Pten/Rb-* and *Akap12/Rb-*null prostate lesions expressed as log_2_ fold changes using FPKM to normalize for gene expression changes. Expression differences for *Akap12*, *Akt2* and *Pten* between the *Pten/Rb-* and *Akap12/Rb-*null tumors were significant (*p* < 0.05). **E** Analysis of relative Akt isoforms and poS473 levels were analyzed in prostate lysates from 4 month-old WT, *Akap12/Rb-* or *Pten/Rb-*null Tg by IP using isoform-specific Abs followed by IB for either Akt^poS473^ or Akt isoforms. **F** IB analysis of Akt substrates in prostate lysates from WT, *Pten/Rb-* and *Akap12(“A12”)/Rb-*null mice using an Ab specific for the canonical phosphorylation motif, RXXS^po^/T^po^.

Although the loss of *AKAP12* is less frequent than *PTEN* loss in primary PC (10% vs. 31% in the TCGA dataset), *PTEN* or *AKAP12* losses statistically co-occur with *RB1* loss (Supplementary Table S1), and these combinations show statistical significance in predicting disease-free survival (DFS) using TCGA datasets (Fig. 1C). AKAP12 is thought to attenuate oncogenic Src signaling by scaffolding pools of Src to lipid rafts, away from integrin/FAK/growth factor receptor-rich plasma membrane sites [13]. Indeed, the inducible upregulation of Akap12 in MLL[Tet^OFF^-SSeCKS/Akap12] PC cells [7] decreased relative phosphotyrosyl-p85α and AKT^poS473^ levels without affecting total AKT, p85α or PTEN levels (Fig. 1D). This correlates with a statistical co-occurrence between *AKAP12* loss and either increased levels of the SFK member, *LYN* (copy number gain, transcriptional upregulation) or Src^poY416^, a shared marker of SFK activation [62] (Fig. 1E). This is consistent with the notion that *LYN* promotes PC progression and mPC formation [16, 63–65], whereas *FYN*, whose levels trend towards mutual exclusivity with *AKAP12* loss (Fig. 1E), is thought to promote progression of neuroendocrine PC [66]. Yet, whereas both *Pten/Rb-* and *Akap12/Rb-*null prostate lesions exhibit Akt activation (Fig. 2A) [3, 40], their mPC progression profiles differ (Table 1): *Pten/Rb-*null mice develop aggressive prostatic adenocarcinomas associated with systemic metastases, whereas *Akap12/Rb-*null mice develop HG-PIN plus local, indolent lymph node metastases. This suggests that PTEN or AKAP12 control divergent AKT oncogenic progression pathways, with the latter likely depending more on SFK roles. Indeed, the majority of primary PC cases with *PTEN* loss are distinct from those with *SRC* or *LYN* gain (Supplementary Fig. S1A). Thus, “AKT activation” in the context of RB loss is not sufficient for progression to adenocarcinoma.

**Table 1:** Pathology of prostate lesions in transgenic CaP models.

Genotype	Prostate lesion	Metastasis	AKT^poS473^
WT	None	No	−
Pb4-Cre:*Rb*^*fl/fl*^	Hyperplasia	No	−
*Akap12*^*−/−*^	Hyperplasia	No	+
Pb4-Cre:*Pten*^*fl/fl*^	PIN	Rare	+
*Akap12*^*−/−*^*;*Pb4-Cre:*Rb*^*fl/fl*^	HG-PIN	Yes (local LN)	+
Pb4-Cre:*Pten*^*fl/fl*^*;Rb*^*fl/fl*^	Adenocarcinoma	Yes (lung, liver, LN, bone)	+

*Pb* probasin, *PIN* prostatic intraepithelial neoplasia, *HG* high-grade, *LN* lymph node.

### Preferential dependence on AKT2 in PTEN-deficient PC cells

We addressed whether differential Akt isoform usage/dependence might account for the varying mPC progression profiles of the two transgenic models. AKT isoforms exert different effects on the survival of PC cell lines [32, 38, 39, 67, 68], and importantly, Chin et al. [38] showed that PTEN-deficient human LNCaP cells show a greater reliance on AKT2 for maintenance and survival in anchorage-independent growth conditions. Analysis of the three AKT isoforms, AKT1, AKT2, and AKT3, identified a statistically significant increase in only AKT2 in mPC in three human Oncomine datasets [14, 25, 69] (Supplementary Fig. S1B), suggesting a more important role for AKT2 in mPC progression. IB analysis of prostate lysates from 12 week-old WT, *Akap12/Rb-*, or *Pten/Rb-*null mice indicated that relative Akt^poS473^ levels were increased in *Akap12/Rb-* and *Pten/Rb-*null lesions compared to levels in WT prostates (Fig. 2A). Ser473 is phosphorylated by mechanistic target-of-rapamycin complex 2 (mTORC2) [70] and PI3K [71], and is required to potentiate AKT serine/threonine kinase activity [30]. In addition, the relative increase of the pan-AKT substrate, Pras40^poT246^, suggests similar levels of overall Akt activation in *Akap12/Rb-* and *Pten/Rb-*null compared to WT prostates (Fig. 2A). In contrast, the relative level of Akt^poT308^, a residue phosphorylated by PDK1 [72] but not required for AKT’s ability to phosphorylate downstream targets [70], was elevated only in *Pten/Rb*-null prostates, when normalized to β-actin as a loading control. However, we found no change in relative PDK1 protein or activation levels in WT, *Akap12/Rb-*, or *Pten/Rb-*null prostates (Fig. 2B). As we showed previously [19], the loss of *Akap12* alone was sufficient to induce activated Akt in all four prostatic lobes (Fig. 2C) using an Ab that recognizes poS473/474 shared by AKT1/2. Compared to WT prostates, higher Akt1/2^poS473/474^ levels were also detected in *Akap12/Rb-* and *Pten/Rb-*null prostates (Fig. 2C). There was increased nuclear signal in *Pten/Rb-*null adenocarcinomas (Fig. 2C; bottom), consistent with a previous report of localization of AKT1 in the cytoplasm and AKT2 in the nucleus of PC-3 cells [39]. Moreover, whereas total Akt1 protein levels were similar in all three prostate genotypes, the relative levels of Akt2 and Akt3 were increased in *Pten/Rb-*null prostates (Fig. 2A).

RNA-seq analysis showed no overall changes in *Akt1* and *Akt3* levels between *Akap12/Rb-* and *Pten/Rb-*null prostates, compared to an upregulation of *Akt2* RNA in the *Pten/Rb-*null prostates (Fig. 2D). To assess the relative activation levels of AKT isoforms, AKT isoform proteins were immunoprecipitated using isoform-specific Abs and the pull-downs probed for Akt^poS473^ (Fig. 2E). The relative activation level of Akt1 was similar in *Akap12/Rb-* and *Pten/Rb-*null prostates whereas *Pten/Rb-*null prostates showed increases in Akt2 and Akt3 activation levels, correlating with increased protein levels (Fig. 2A).

Next, we probed these tissue samples with an Ab that detects AKT canonical substrates based on the shared phosphorylated motif, RXXS^po^/T^po^ (Fig. 2F). Whereas the *Akap12/Rb-*null lysates had quantitative differences in the levels of several substrates compared to WT lysates, the *Pten/Rb*-null lysates also had qualitative differences, suggesting the targeting of unique substrates. This finding is consistent with the notion that different AKT isoforms may predominate in the two Tg PC models.

**Fig. 3:**
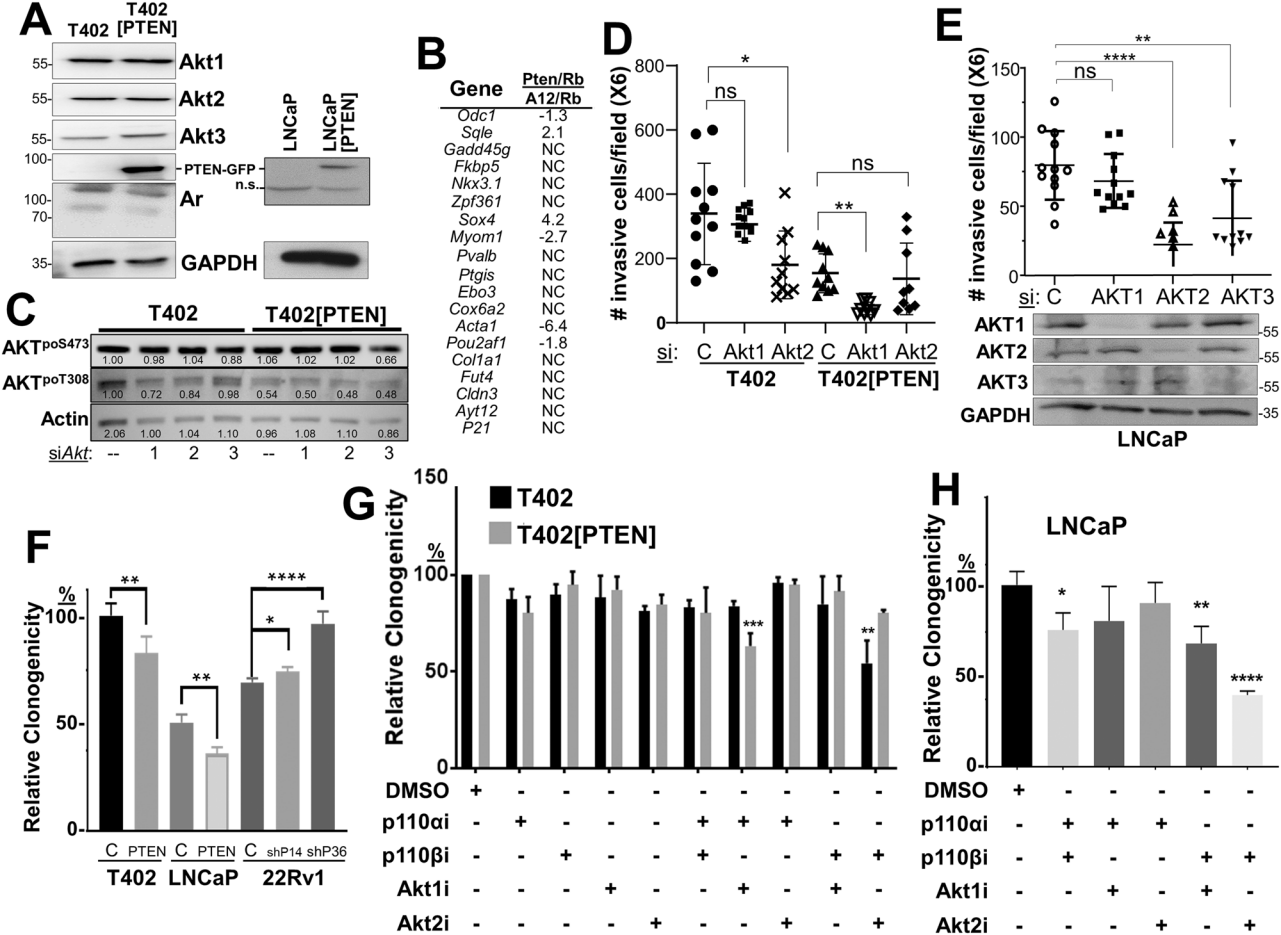
PTEN status controls invasiveness and survival through PI3K-p110 and AKT isoforms. **A** IB analysis of T402 or T402[PTEN], or LNCaP or LNCaP[PTEN] cell lysates showing that PTEN-GFP re-expression does not alter Akt isoform or Ar expression; n.s. non-specific. **B** Relative fold changes in the transcription levels of 19 AR-regulated genes from the RNA-seq analysis in Fig. 2D, comparing *Pten/Rb-*null vs. *Akap12/Rb-*null tumors. **C** IB showing the effect of Akt isoform knockdowns on relative AKT^poS473^ and AKT^poT308^ levels in T402 and T402[PTEN] cells. Matrigel invasiveness of T402 or T402[PTEN] (**D**), or LNCaP (**E**) treated with control (“C”) or AKT isoform siRNAs. **E**, *bottom* IB analysis of AKT isoforms after siRNA treatment. **F** Relative clonogenic survival of isogenic PC pairs, in which parental (“C”) cells are transduced with either PTEN-GFP (“PTEN”) or sh*PTEN* clones (“shP14” or “shP36”). Relative clonogenic survival of T402 or T402[PTEN] (**G**), or LNCaP (**H**) cells treated with PI3K-p110 and/or AKT isoform inhibitors (vs. DMSO vehicle alone). For panels C-G, ns not significant, *P < 0.05, **P ≤ 0.01, ***P ≤ 0.001, ****P ≤ 0.0001 (*n* = 12).

**Fig. 4:**
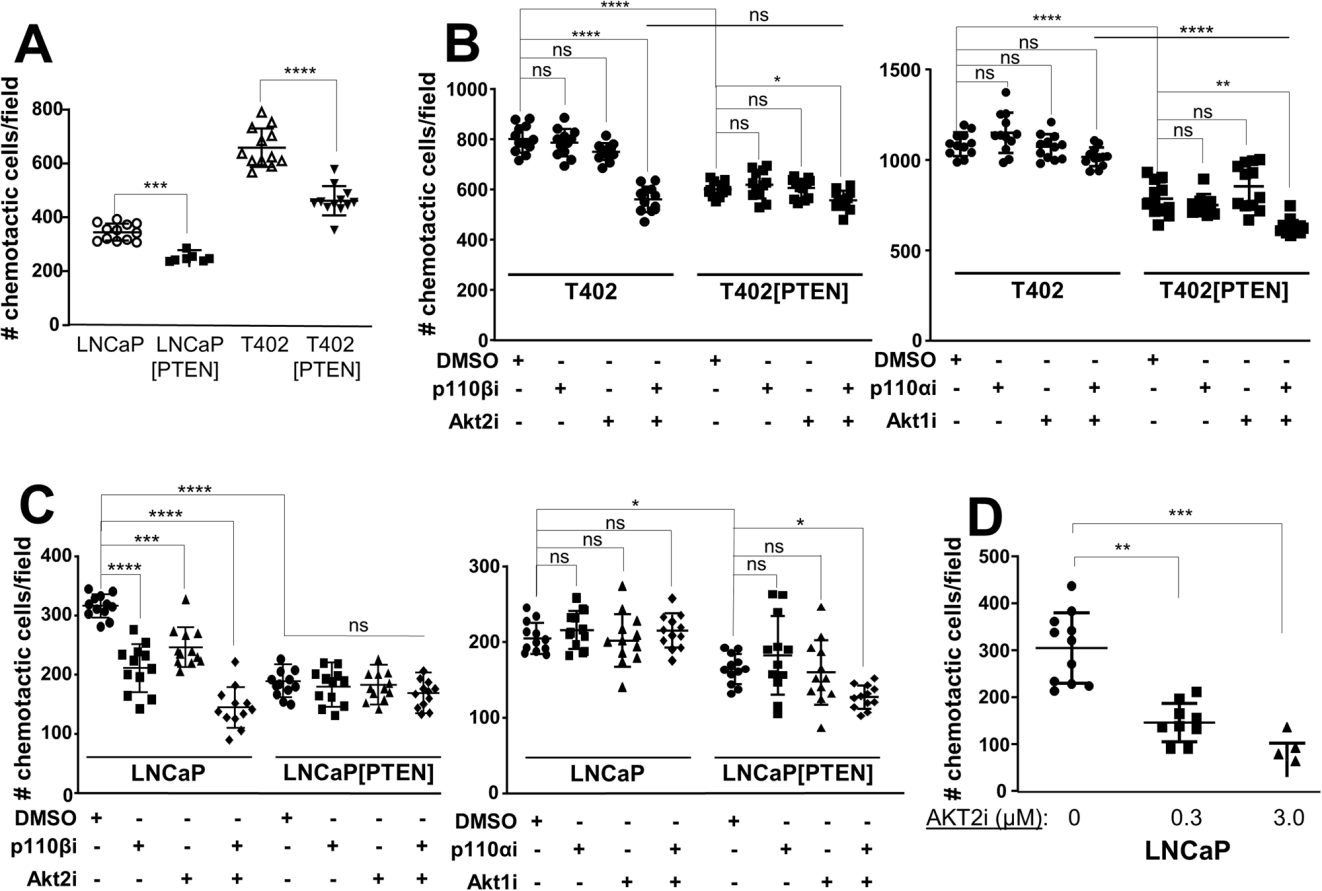
PTEN controls chemotaxis through PI3K-p110 and AKT isoforms. **A** The chemotactic abilities of LNCaP vs. LNCaP[PTEN] or T402 vs. T402[PTEN] were assessed using transwell assays with serum added to the lower chamber. The effect of PTEN re-expression, p110 and/or AKT isoform inhibitors on T402 (**B**) or on LNCaP isogenic pair (**C**) chemotaxis was assessed, as was the effect of increasing AKT2i on LNCaP (**D**). ns not significant. *P < 0.05, **P ≤ 0.01, ***P ≤ 0.001, ****P ≤ 0.0001.

We then addressed how PTEN status controls PC oncogenic growth by re-expressing PTEN-GFP (vs. GFP alone in controls) in LNCaP or T402 cells (Table 2, Fig. 3A), the latter derived from a murine *Pten/Rb-*null adenocarcinoma [40]. As well, we produced an isogenic pair of PTEN-positive 22Rv1 cells expressing shPTEN or scrambled (control) shRNA (Supplementary Fig. S2D). PTEN re-expression in T402 cells neither changed protein levels of Akt isoforms or Ar (Fig. 3A), the relative expression of an Ar-regulated 19-gene panel (Fig. 3B), nor proliferation in 2D conditions with androgen-containing media (Supplementary Figs. S2A–C). Importantly, relative AKT^poS473^ levels were unchanged after knockdown of each *Akt* isoform in T402 cells (*Akt* knockdown levels are shown in Supplementary Fig. S3E), and only slightly decreased in T402[PTEN] cells with *Akt3* knockdown (Fig. 3C). Relative AKT^poT308^ levels were decreased slightly by Akt1 or Akt2 loss in T402 cells, whereas these levels were decreased by the expression of PTEN about twofold relative to siControl in T402 cells, with no effect by Akt isoform knockdown. These data indicate that under 2D growth conditions, PTEN neither controlled Akt isoform abundance nor relative poS473 levels. Consistent with its role as a tumor suppressor, PTEN re-expression decreased relative PC invasiveness (Fig. 3D), clonogenicity (Fig. 3F), and chemotaxis (Fig. 4A, C), whereas PTEN knockdown in isogenic 22Rv1 cells increased clonogenicity (Fig. 3F).

**Table 2:** CaP cell line models.

Model	Species	PTEN	AR	AKT^poSer473, a^
T402 (*Pten, Rb-*negative)	Mouse	Deleted	+	++
T402[PTEN]	Mouse	+	+	Low (in 3D)
LNCaP^b^	Human	del/mut**	+	++
LNCaP[PTEN]	Human	+	+	Low (in 3D)
22Rv1	Human	+	H874Y	Low (in 3D)
22Rv1[shPTEN]	Human	Low	H874Y	+

^a^Based on IB, relative to total AKT1 protein levels.

^b^One copy deleted, one copy with a truncation mutation.

Based on the increase in Akt2 expression and activation in the more metastatic *Pten/Rb-*null model (Fig. 2A, E), we asked if the knockdown of Akt2 would inhibit in vitro parameters of metastatic growth, using transwell assays for chemotaxis or Matrigel invasion, or by assaying for survival using either clonogenic or anoikis assays. AKT knockdowns in T402 and LNCaP cells were isoform-specific (Fig. 3D, lower panel; Supplementary Fig. S3E). The knockdown of Akt2, but not Akt1, in the *Pten*-negative T402 PC cells (Supplementary Fig. S3E) decreased invasiveness (Fig. 3D). In contrast, the re-expression of *PTEN*, which decreased the invasiveness of control T402 cells, switched dependence from Akt2 to Akt1. Similarly, the knockdown of either AKT2 or AKT3, but not AKT1, inhibited LNCaP invasiveness (Fig. 3E). PTEN re-expression decreased the invasiveness of LNCaP cells to the limits of detection (<20 cells/field), thereby making it impossible to assess the effects of AKT isoform knockdown. Although AKT3 levels were quite low in LNCaP cells (requiring IP from 0.5 mg of lysate protein followed by IB), knockdown caused a decrease in invasiveness (Fig. 3E), strongly suggesting that AKT3 promotes invasiveness in these cells. Taken together, these data identify critical roles for AKT2 and AKT3 in the invasiveness of PTEN-deficient PC, and that upon PTEN re-expression, reliance on AKT1 increases. They also indicate that relative AKT^poS473^ levels, typically taken as a gauge of AKT activation levels, are insufficient to describe AKT isoform dependency relative to specific premetastatic growth/motility parameters.

### Role of PI3K-p110 and AKT isoforms in PTEN-regulated survival and chemotaxis

Because PTEN-negative tumor cells have been reported to depend more on p110β for oncogenic growth [33, 56], we next assessed how PTEN expression affected clonogenic survival of the PC isogenic pairs, and if PTEN affected sensitivity to small molecule inhibitors of PI3K-p110 and AKT isoforms, or after knockdown of p110/AKT isoforms. PTEN re-expression decreased the relative clonogenic survival of LNCaP or T402 cells, whereas PTEN knockdown increased survival of 22Rv1 cells (Fig. 3F). To address the role of PI3K and AKT isoforms in controlling clonogenic survival, we first identified concentrations of p110α, p110β, AKT1 and AKT2 inhibitors that had minimal effect on the 2D proliferation of PC lines but were significantly above the IC50’s reported for each drug (examples in Supplementary Figs. S2E and S4D). Importantly, we sought to show that the effects of the isoform-specific inhibitory drugs mimicked what we found with isoform-specific AKT and/or p110 si/shRNAs. Only the combination of p110βi and Akt2i significantly reduced the number of colonies in T402, whereas in T402[PTEN] cells, sensitivity changed to a combination of Akt1i and p110αi (Fig. 3G). Similar results were seen in LNCaP (Fig. 3H) and with other PTEN-positive or -negative human PC cells lines (Supplementary Fig. S3A, B), or when combining knockdown of p110 (Supplementary Fig. S3F) and AKT isoforms (Supplementary Fig. S3C, D). Thus, these data suggest a plasticity with which PTEN directs survival dependency through both p110α and AKT1, whereas PTEN-deficient cells depend on both p110β and Akt2. Akt3 was not included because of the lack of Akt3-specific inhibitors.

We then analyzed the PC isogenic cell panel for the effects of PTEN on chemotaxis. 22Rv1 cells, which are very poor at chemotaxis even if PTEN is knocked down, were omitted. The reintroduction of PTEN significantly reduced chemotaxis in LNCaP and T402 (Fig. 4A). We next asked if the differential PI3K and AKT drug sensitivities observed in the clonogenic assays also affected chemotaxis. Chemotaxis in T402 was inhibited by the combination of AKT2 and p110β inhibitors (Fig. 4B; Supplementary Fig. 4B). PTEN re-expression abrogated most of the combined effect of AKT2i plus p110βi. As was observed in the clonogenic assays, T402 chemotaxis was not inhibited by p110αi and/or AKT1i, whereas in T402[PTEN], chemotaxis was sensitive to the combination of p110αi and Akt1i. Similar results were observed in LNCaP using isoform-inhibitory drugs (Fig. 4C) or RNAi (Supplementary Fig. S4A), noting that 0.3 μM AKT2i was insufficient to inhibit LNCaP 2D proliferation (Supplementary Fig. S2E) but sufficient to inhibit chemotaxis (Fig. 4D).

Zhang et al. [37] showed that the dependence of PTEN-deficient BT549 (breast) and PC3 (prostate) cancer cells on p110β was likely due to the selective binding by CRKL to p110β, facilitated by Src-phosphorylated p130Cas. This correlated with increased suppression of PTEN-deficient tumor growth by combining p110β and Src inhibitors. We recapitulated these findings in LNCaP and T402 cells using both our p110βi and the Src inhibitor, Saracatinib (Supplementary Fig. S4C). Additionally, *Crkl* RNA levels are roughly 4.2-fold higher in *Pten/Rb-*null than in *Akap12/Rb*-null tumors (Supplementary Fig. S4D). These data strengthen the notion that the p110β/AKT2 pathway activated in the absence of PTEN is separate from the Src/p110α/AKT1 pathway activated in the absence of AKAP12 (Fig. 1D).

We then attempted gain-of-function experiments using constitutively-active (CA) AKT1^S473D^ or AKT2^S474D^. In a previous study, CA-AKT1^S473D^, but not CA-AKT2^S474D^, rescued the phosphorylation of the mTORC2-dependent substrate, ATP-citrate lyase, in brown adipocytes [41]. The stable expression of AKT1^S473D^ or AKT2^S474D^ in T402 cells equally increased the number and abundance of phospho-AKT substrates irrespective of PTEN status (Supplementary Fig. S4E), validating the notion that they encode CA kinase variants. However, there was no distinction in the ability of either CA-AKT isoform to increase chemotaxis in either T402 or T402[PTEN] cells (Supplementary Fig. S4F). We found similar results using AKT1 or AKT2 constructs fused to an N-terminal myristylation domain known to potentiate associated kinase activity [73, 74], namely, no distinction in the ability to induce phospho-AKT substrates and chemotaxis (data not shown). Thus, it is likely that in LNCaP or T402 cells, the CA mutants cannot differentiate AKT1- vs. AKT2-specific functions.

### SMAD4 loss as a marker of p110β/AKT2 dependence in PTEN-deficient PC cells

**Fig. 5:**
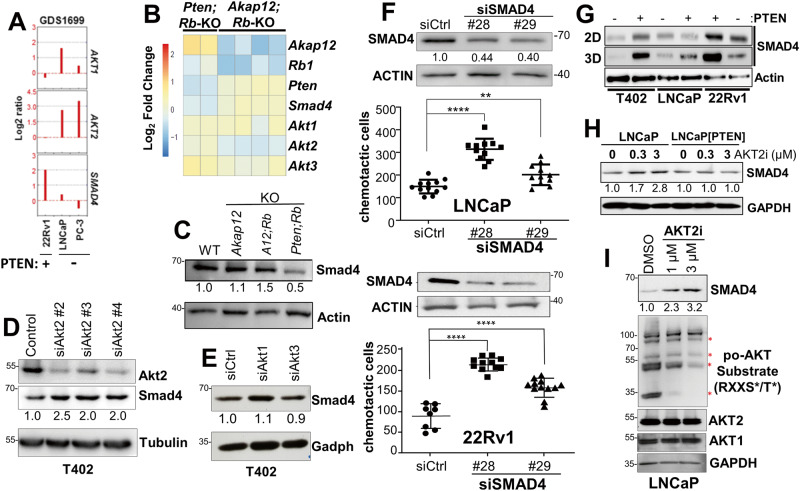
Inverse relationship between AKT2 and SMAD4 expression. **A** The relative levels of *AKT1*, *AKT2*, and *SMAD4* RNA levels vs. PTEN status in human PC cell lines assessed in NCBI GEO dataset GDS1699. Heat map of RNA-seq data comparing *Smad4* and *Akt2 RNA* levels (**B**) or Smad4 protein levels (**C**) from *Pten/Rb-* and *Akap12/Rb-*null (KO) prostate lesions. Knockdown of Akt2 (**D**), but not Akt1 or Akt3 (**E**) in T402 cells correlates with increased Smad4. **F** SMAD4 knockdown in LNCaP and 22Rv1 (analyzed by IB in the top panels) correlates with decreased chemotaxis (bottom panels). Numbers under IBs in panels C-F describe relative SMAD4 protein levels (vs. protein loading controls). **P ≤ 0.01, ****P ≤ 0.0001. **G** IB analysis for SMAD4 or β-Actin in T402, LNCaP or 22Rv1 cell pairs isogenic for PTEN that were grown in 2D vs. 3D conditions. **H** Treatment (24 h) with AKT2i induces SMAD4 in LNCaP but not in LNCaP[PTEN] cells. **I** Induction of SMAD4 by AKT2i in LNCaP correlates with decreased levels of AKT substrates, assessed by IB using canonical AKT substrate motif Ab (RxxS^po^/T^po^), but no changes in total AKT1 or AKT2 protein levels.

Previous data showed that *Smad4* loss potentiates PC metastasis formation in *Pten*^*PE:−/−*^ mice [61]. We analyzed whether Smad4 might serve as a marker of metastatic progression that could differentiate the aggressive *Pten/Rb-*null PC model from the indolent *Akap12/Rb-*null HG-PIN model. *SMAD4* RNA levels inversely correlated with *AKT2*, but not *AKT1*, RNA levels in human PC cell lines (Fig. 5A) and when comparing *Akap12/Rb*- vs. *Pten/Rb*-null prostate lesions (Fig. 5B). This corresponded to lower Smad4 protein levels in the more metastatic *Pten/Rb*-null tumors (Fig. 5C) and in human metastatic PC (Supplementary Fig. S5). Smad4 protein levels were increased 2- to 2.5-fold by the knockdown of Akt2 (Fig. 5D) but not by the knockdown of Akt1 or Akt3 (Fig. 5E; the efficacies of the si*Akt1* and si*Akt3* are shown in Supplementary Fig. S3E). In LNCaP and 22Rv1 cells, the knockdown of SMAD4 using two different siRNAs led to a significant increase in chemotaxis (Fig. 5F). Moreover, SMAD4 levels in T402, LNCaP and 22Rv1 cells grown in 3D were consistently higher than their PTEN-deficient isogenic mates, and these increases were higher in cells grown in 3D vs. 2D (Fig. 5G). Treatment with AKT2i induced SMAD4 expression in LNCaP (Fig. 5H, I) but not in LNCaP[PTEN] cells (Fig. 5H), and this correlated with decreased abundance of po-AKT substrates but not total AKT1 or AKT2 (Fig. 5I), confirming the efficacy of AKT2i. In contrast, concentrations of AKT1i or p110αi that did not inhibit LNCaP 2D proliferation or survival (Supplementary Figs. S2E and S3A) also failed to decrease LNCaP chemotaxis (Fig. 4C), strengthening the role of AKT2 in controlling chemotactic motility of PTEN-negative PC cells.

**Fig. 6:**
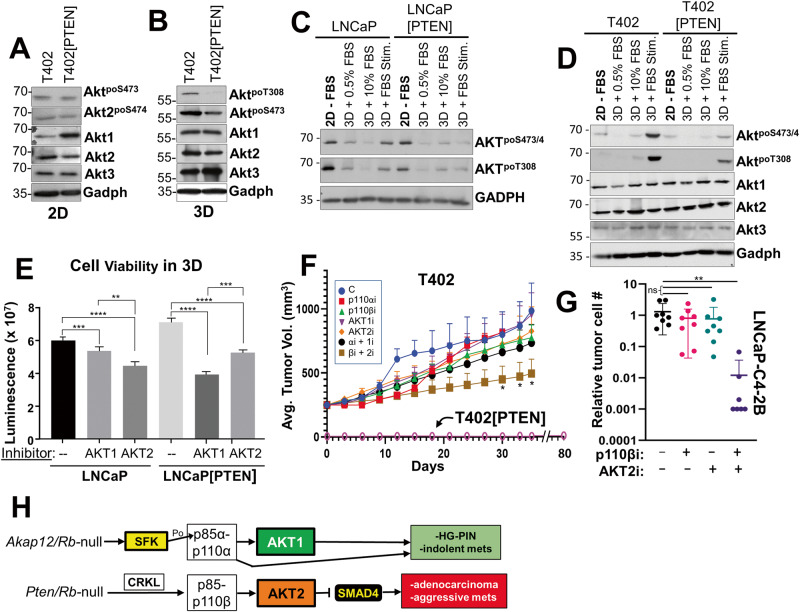
3D growth potentiates reduction of AKT activation by PTEN. IB analysis of Akt isoform and phosphorylation levels in T402 and T402[PTEN] incubated in 2D (**A**) or 3D (**B**) conditions for 24 h. IB analysis showing Akt isoform and phosphorylation levels in LNCaP or LNCaP[PTEN] (**C**), or T402 or T402[PTEN] (**D**) cells grown under the following conditions: (i) 2D for 18 h in media without serum (“−FBS”), (ii) 3D growth for 18 h in media with 0.5% FBS, (iii) 3D for 18 h in media with 10% FBS, iv) 3D for 18 h in media with 0.5% FBS, followed by 30 min of 10% FBS (“3D + FBS Stim.”). **E** Cell viability, assessed using CellTiter-Glo 3D Cell Viability Assays, as described in Materials and methods, of LNCaP and LNCaP[PTEN] grown for 48 h in anoikic (3D) growth conditions in the presence or absence of AKT1i or AKT2i (*n* = 8). **F** Tumor growth in male SCID mice (6/group) injected orthotopically with T402 or T402[PTEN] after 28 days of daily treatment with vehicle (“C”) or individual or combinations of p110 or AKT isoform inhibitors. *P < 0.05. **G** Metastatic dissemination to the liver in male SCID mice (5/group) 3 weeks after subcutaneous injection with LNCaP-C4-2B[luc/GFP], with daily treatments of vehicle (“−”), p110βi, AKT2i or p110βi + AKT2i. ns not significant; **P < 0.01. **H** Tg model for differential reliance on p110 and Akt isoforms relative to PC progression. The loss of *Akap12* in the context of *Rb* loss results in activation of SFK (Src or LYN), which then favors the activation of and dependence on PI3K-p110α (through the direct phosphorylation of p85α) and Akt1. This results in HG-PIN and the dissemination of indolent metastatic cells to local lymph nodes. In contrast, the loss of *Pten* and *Rb* favors activation of and dependence on PI3K-p110β (through upregulation and association with Crkl) and Akt2 (and possibly Akt3). PI3K-p110β/Akt2 signaling suppresses Smad4 expression, resulting in the formation of adenocarcinomas and aggressive, systemic metastases.

We next compared how PTEN re-expression affected AKT signaling in 2D vs. 3D growth. This is because we previously showed that activated Src had a maximal ability to activate AKT in 3D growth conditions [75] and because the effect of AKT2 on LNCaP survival was manifest in 3D, but not in 2D growth [38]. Interestingly, the re-expression of PTEN did not significantly reduce activated AKT (relative AKT^poSer473^ or AKT2^poSer474^ levels, the latter using an Ab specific for activated AKT2) in T402 grown in 2D (Fig. 6A). In contrast, PTEN-mediated reduction in relative AKT^poT308^ levels were observed in cells grown in 3D (suspension in methylcellulose) (Fig. 6B). We then analyzed how PTEN affected the ability of serum to induce AKT activation in 2D vs. 3D conditions. While the overnight growth in 3D with serum had minimal to no ability to activate AKT, a 30 min treatment of FBS (“3D + FBS Stim.”) to serum-starved (“3D + 0.5%FBS”) LNCaP or T402 cells induced more relative AKT^poSer473/474^ and AKT^poT308^ in PTEN-negative cells than in PTEN re-expressing cells (Fig. 6C, D). We next determined if PTEN controlled survival under anoikis conditions was through a greater dependence on AKT1 or AKT2. LNCaP and LNCaP[PTEN] cells were grown in non-adherent conditions (48 h on agarose-coated plates) while being treated with DMSO, AKT1i, or AKT2i, followed by quantification of cell viability. LNCaP viability was more dependent on AKT2, whereas viability of LNCaP[PTEN] cells was more dependent on AKT1 (Fig. 6E). Taken together, these data strongly suggest that PTEN suppression of AKT activation is potentiated under 3D conditions, exemplified here by increased survival under anchorage-independent conditions but also by other 3D conditions shown earlier such as invasiveness and chemotaxis.

### Therapeutic targeting of PTEN-negative PC requires combining PI3K-p110β and AKT2 inhibitors

We addressed how targeting p110 and/or AKT isoforms affected the progression of primary orthotopic tumors and the establishment of spontaneous metastases in SCID male mice. Consistent with PTEN’s tumor suppressor function, the orthotopic injection of T402[PTEN] cells (prostatic anterior lobe) failed to yield growing tumors after 80 days (Fig. 6F). Individual inhibitors for p110α, p110β, AKT1 or AKT2, or the p110α/AKT1 combination, slightly decreased tumor growth from days 12-18 of drug treatment in comparison to vehicle controls, however, none of these translated to statistically significant effects at day 35. In contrast, treatment with the combination of p110β/AKT2 inhibitors showed statistically significant tumor suppression over controls. Moreover, the p110β/AKT2 inhibitor combination resulted in a statistically significant decrease in metastatic colonization by LNCaP-C4-2B[luc/GFP] cells, as assessed by *Alu*-specific qPCR as described previously [52] (Fig. 6G). These data extend the notion that p110β and AKT2 are the major drivers of tumor and metastasis formation in the absence of PTEN.

## Discussion

The high frequency of *PTEN* loss in primary PC as well as transgenic prostate models showing that *Pten* loss is sufficient to induce a tumor-prone state for PC initiation and progression has been premised on the assumption that this translates to activation of oncogenic PI3K-AKT signaling [21, 57, 76]. However, our comparison of two Tg PC models, *Pten/Rb*-null vs. *Akap12/Rb*-null, which share similar levels of Akt activation plus Rb loss, yet have different outcomes regarding primary PC and mPC progression, strongly suggests that “AKT activation” alone is insufficient to explain these differences. Based on growing evidence showing that the three AKT isoforms play differing roles in the progression of some cancers, including PC [38, 39], the differing disease phenotypes in our two transgenic models could be explained by the differential activation and preferential dependence on specific AKT isoforms. Although previous studies compared the possible roles for AKT isoforms after AR re-expression in PC-3 cells [77] or by comparing AR-positive vs. -negative human PC cell lines [78], ours is the first study to analyze the effect of PTEN re-expression or knockdown in isogenic cell lines. Our data strongly suggest that the loss of *PTEN* induces mPC through a PI3K-p110β/AKT2-mediated pathway. In contrast, the loss of AKAP12, which normally scaffolds Src and attenuates Src-induced PI3K/AKT activation, correlates with weaker oncogenic progression signaling through p110α and AKT1 (Fig. 6H).

We show evidence suggesting that PC is marked by at least two relatively non-overlapping driver pathways, both of which impact PI3K/AKT signaling: *PTEN* loss and Src-family kinase activation. This divergence is underlined by TGCA Firehose data showing that the roughly one-third of primary PC cases exhibiting gene amplifications in the SFK genes, *SRC, LYN*, and *FYN*, have little overlap with those suffering *PTEN* deletions (Supplementary Table S1). Indeed, SFK mutations known to induce oncogenic activation are extremely rare in PC (0/491 cases in TCGA Firehose). Thus, our development of the *Akap12/Rb-*null transgenic PC model was meant to genocopy the oncogenic activation of SFK, given AKAP12’s role as a Src scaffolding protein [13] and our finding that *AKAP12* loss and *LYN* gain co-occur in TCGA datasets.

Our data indicate that *Pten/Rb-*null PC lesions express higher protein and activation levels of Akt2 than HG-PIN lesions from *Akap12/Rb-*null mice. Although higher Akt3 protein levels were detected in *Pten/Rb-*null PC lesions, this increase was not manifest at the RNA level. Importantly, 3D proliferation, Matrigel invasiveness, chemotaxis, clonogenic survival, and anchorage-free (anoikis) survival in human and mouse *PTEN-*negative PC cell lines were more dependent on AKT2 or AKT3 (the latter only in the case of LNCaP invasiveness) than on AKT1, consistent with a previous report [38] showing that 3D spheroid growth of PTEN-negative PC cells relies more on AKT2. However, our data show that AKT2 inhibition on multiple measures of in vitro and in vivo metastatic growth/motility of PTEN-deficient PC cells is potentiated by co-inhibiting p110β. In contrast, PTEN re-expression, although decreasing these measures of metastasis, reversed dependency to p110α plus AKT1. It is noteworthy that AKT2 is known to control other cancer progression biologies such as epithelial-to-mesenchymal transformation in breast cancer [79], breast cancer stem cell self-renewal [80], and lung cancer invasiveness [81].

The synergistic effect of co-inhibiting p110 and AKT isoforms may reflect incomplete inhibition of each part of a linear PI3K-AKT pathway, but it is also consistent with previous studies showing AKT-independent functions for PI3K [82] and PI3K-independent AKT roles in cancer [83].

There is growing appreciation for AKT3’s role in cancer progression [78, 84, 85], and indeed, our data showed that knockdown of AKT3 decreased LNCaP invasiveness. The dearth of knowledge regarding AKT3 in cancer possibly stems from early reports that it is expressed predominantly in the brain, heart, and kidneys [86], whereas AKT1 and AKT2 are expressed ubiquitously [87]. However, all three isoforms are expressed in normal and cancerous prostate tissue [88]. Although AKT3 levels do not increase in clinical mPC vs. primary PC cases, our ability to target this isoform is confounded by a lack of an AKT3-specific small molecule inhibitor.

The notion of therapeutically targeting AKT in cancer has been raised previously [87]. Indeed, pre-clinical [89–92] and clinical [93] studies show therapeutic efficacy in targeting AKT or co-targeting AKT plus the androgen axis/androgen receptor in castration-recurrent PC, yet ours is the first to use isogenic PC cell lines to address how PTEN and AKT isoforms affect metastatic signaling and progression. Importantly, our data not only validate the notion of compensatory plasticity between p110 and AKT isoforms, as described previously [33, 34], they identify a role for PTEN in controlling which p110-AKT isoform pairs serve as drivers. More specifically, in the context of Rb loss, p110β/AKT2 seem to drive aggressive mPC progression whereas p110α/AKT1 drive a more indolent mPC disease. Indeed, Crkl, which seems to drive the activation of p110β in PTEN-deficient cancer cells [37], was significantly increased in PC tumors from *Pten/Rb-*null vs. *Akap12/Rb-*null mice. One possibility for the different mPC disease outcomes might be that these pathways have different effects on AR signaling, based on a report of increased AR activation through increased AKT signaling in PTEN-deficient PC tumors [94]. However, PTEN expression did not affect Ar protein levels or the expression levels of 19 Ar-regulated genes in T402 cells.

In sum, our data indicate that PTEN alters AKT isoform dependency such that metastatic prostate cancer progression likely involves substrates preferred by AKT2 and/or AKT3, but not by AKT1. We posit that mPC progression pathways can be therapeutically targeted using the appropriate combination of p110 and AKT isoform inhibitors based on foreknowledge of *PTEN*, SFK, and *AKAP12* genomic status.

### Supplementary information

Supplementary Fig. S1

Supplementary Fig. S2

Supplementary Fig. S3

Supplementary Fig. S4

Supplementary Fig. S5

Supplementary Table S1

Supplementary Table S2

## Data Availability

Materials and reagents described in the manuscript, including all relevant raw data, will be freely available to any researcher wishing to use them for non-commercial purposes, without breaching participant confidentiality.
