# Correction: PTEN-regulated PI3K-p110 and AKT isoform plasticity controls metastatic prostate cancer progression

**DOI:** 10.1038/s41388-023-02920-2

**Published:** 2023-12-14

**Authors:** Karina A. Miller, Seamus Degan, Yanqing Wang, Joseph Cohen, Sheng Yu Ku, David W. Goodrich, Irwin H. Gelman

**Affiliations:** 1 Department of Cancer Genetics & Genomics, Roswell Park Comprehensive Cancer Center, Elm and Carlton Streets, Buffalo, NY 14209 USA; 2 Department of Pharmacology & Therapeutics, Roswell Park Comprehensive Cancer Center, Elm and Carlton Streets, Buffalo, NY 14209 USA; 3 Dana Farber Cancer Institute and Harvard Medical School, Boston, MA USA; 4 Present Address: American Society of Human Genetics, Rockville, MD 20852 USA; 5 Present Address: Sequence, Inc., Morrisville, NC USA

**Keywords:** Prostate cancer, Cancer genomics

Correction to: *Oncogene* 10.1038/s41388-023-02875-4, published online 24 October 2023

The article PTEN-regulated PI3K-p110 and AKT isoform plasticity controls metastatic prostate cancer progression, written by Karina A. Miller, Seamus Degan, Yanqing Wang, Joseph Cohen, Sheng Yu Ku, David W. Goodrich and Irwin H. Gelman, was originally published electronically on the publisher’s internet portal on 24 October 2023 without open access. With the author(s)’ decision to opt for Open Choice the copyright of the article changed on 28 November 2023 to © The Authors 2023 and the article is forthwith distributed under a Creative Commons Attribution Attribution 4.0 International License, which permits use, sharing, adaptation, distribution and reproduction in any medium or format, as long as you give appropriate credit to the original author(s) and the source, provide a link to the Creative Commons licence, and indicate if changes were made. The images or other third party material in this article are included in the article’s Creative Commons licence, unless indicated otherwise in a credit line to the material. If material is not included in the article’s Creative Commons licence and your intended use is not permitted by statutory regulation or exceeds the permitted use, you will need to obtain permission directly from the copyright holder. To view a copy of this licence, visit http://creativecommons.org/licenses/by/4.0.

