# Supplementary figures and images for: PTEN-regulated PI3K-p110 and AKT isoform plasticity controls metastatic prostate cancer progression
Source: Oncogene. 2023 Oct 24;43(1):22–34. doi: 10.1038/s41388-023-02875-4 (PMC10766561; doi:10.1038/s41388-023-02875-4)

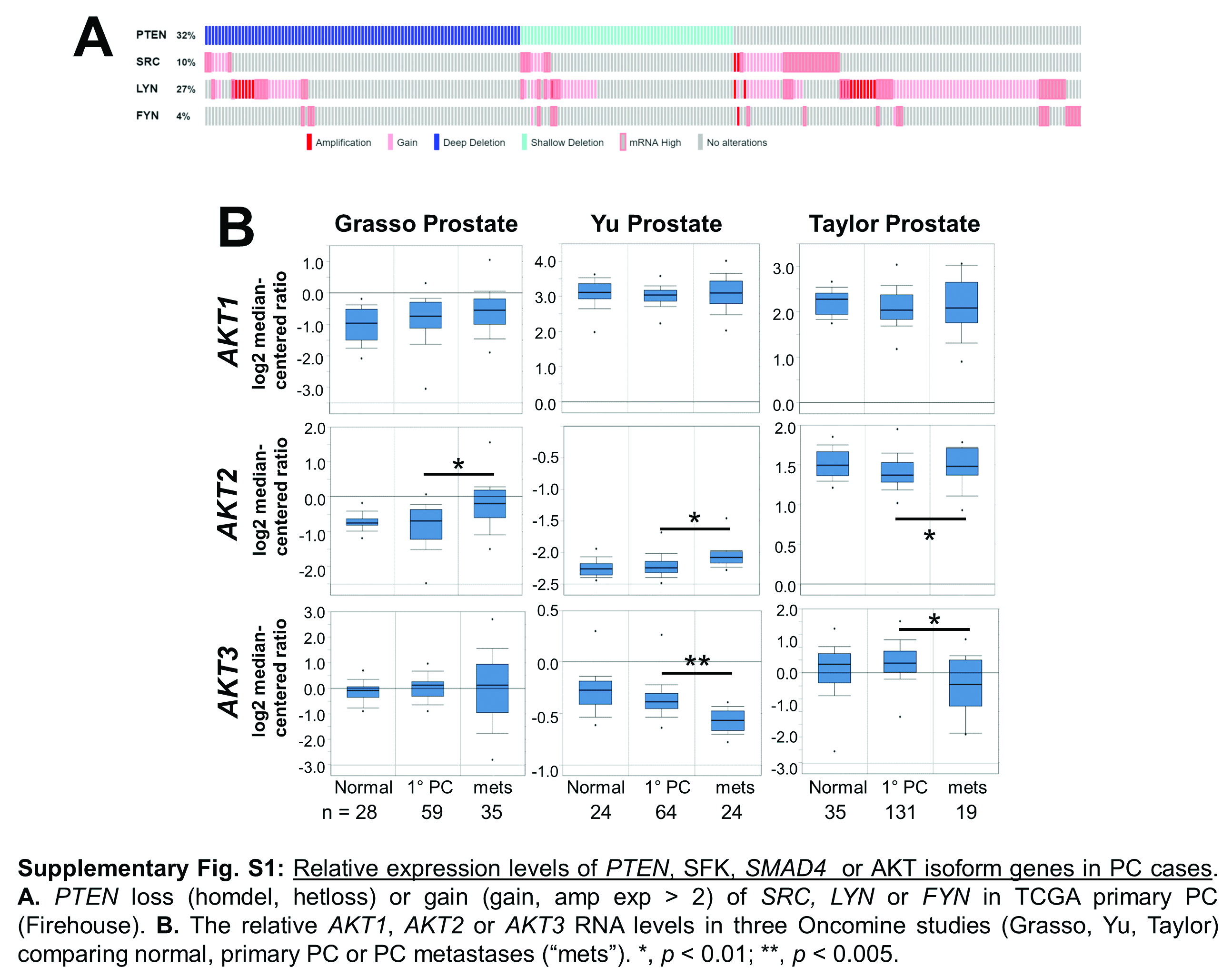

Supplement: Supplementary file 1 — Supplementary Fig. S1 [file 41388_2023_2875_MOESM1_ESM.tif]

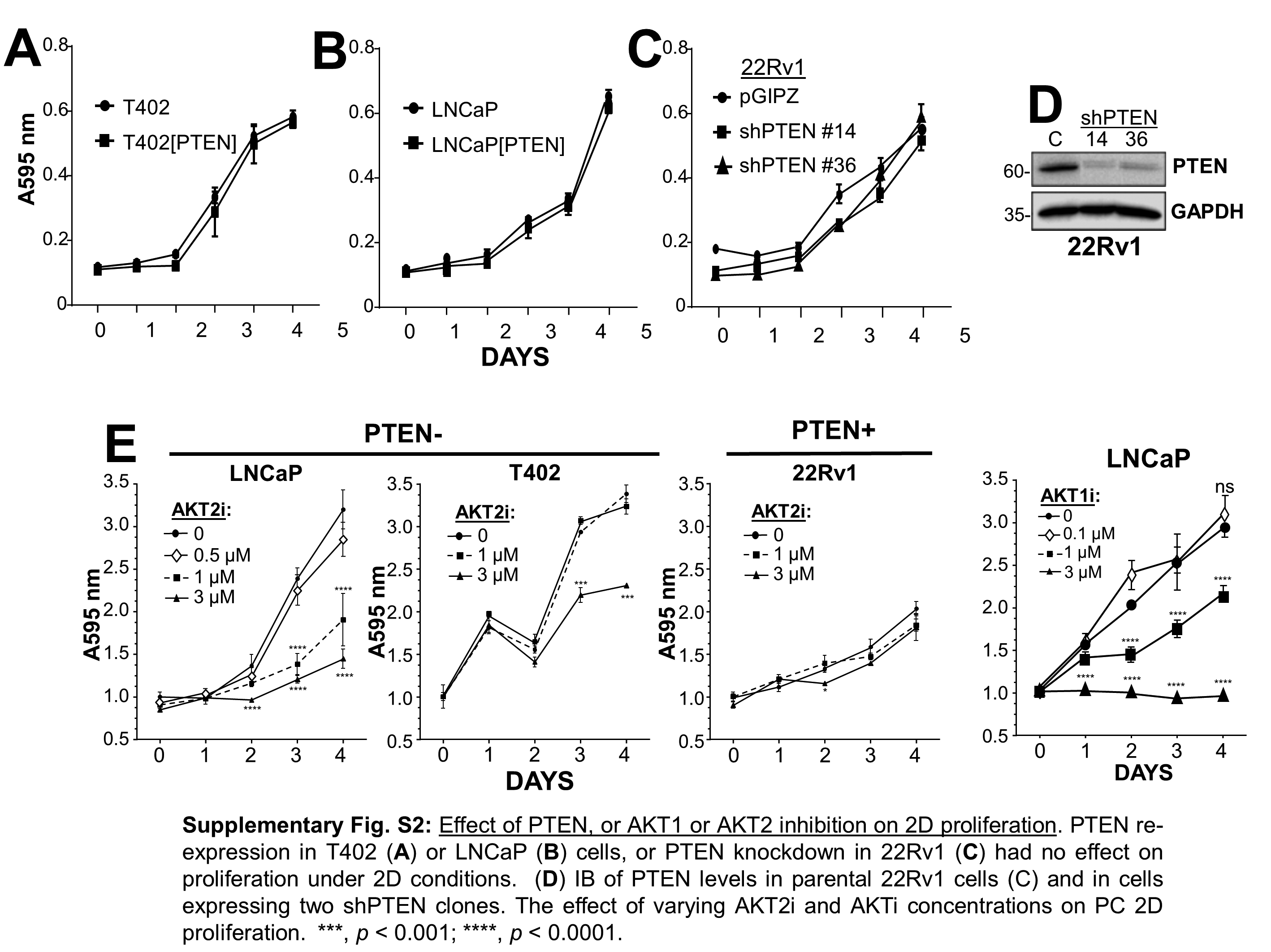

Supplement: Supplementary file 2 — Supplementary Fig. S2 [file 41388_2023_2875_MOESM2_ESM.tif]

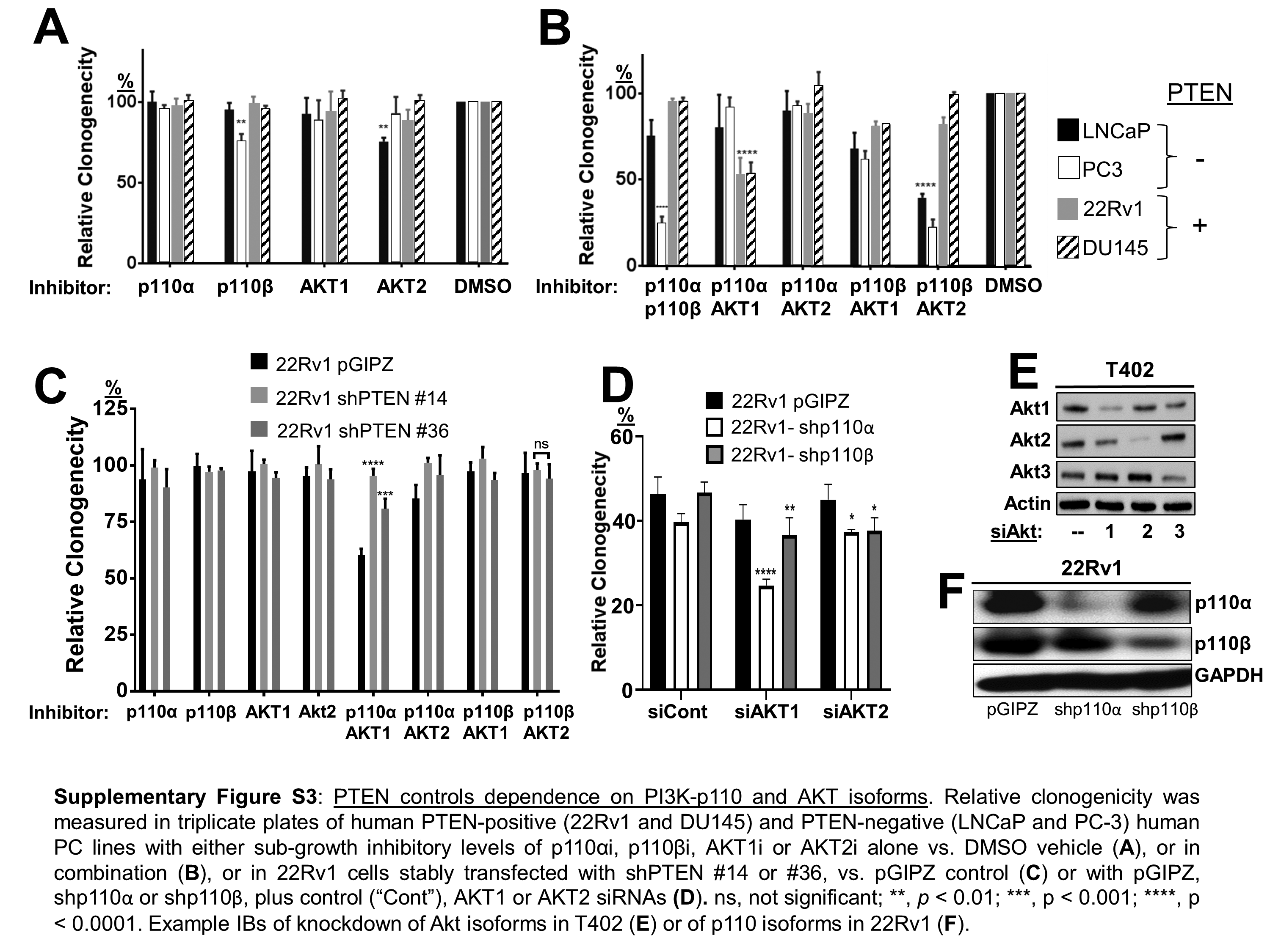

Supplement: Supplementary file 3 — Supplementary Fig. S3 [file 41388_2023_2875_MOESM3_ESM.tif]

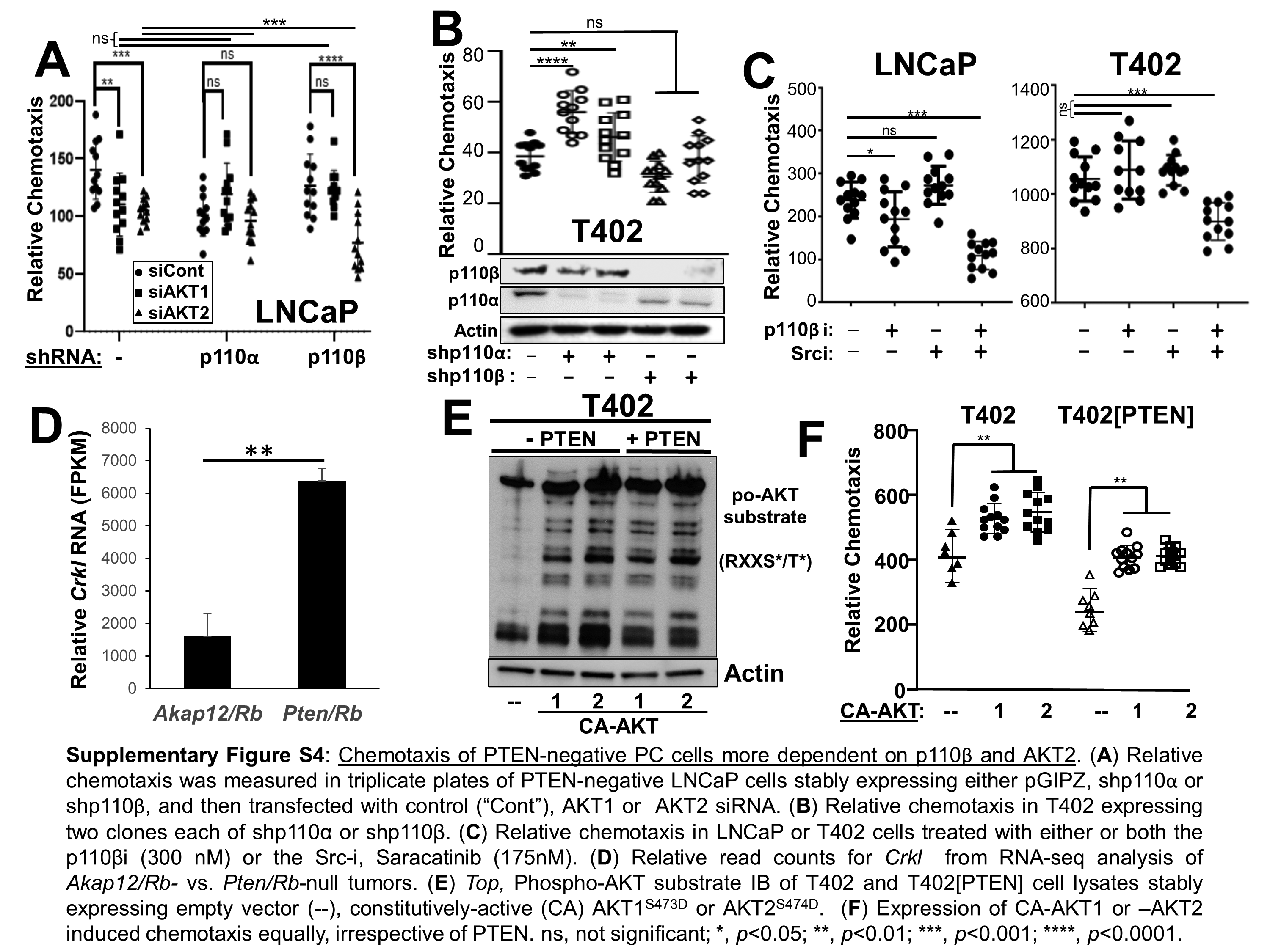

Supplement: Supplementary file 4 — Supplementary Fig. S4 [file 41388_2023_2875_MOESM4_ESM.tif]

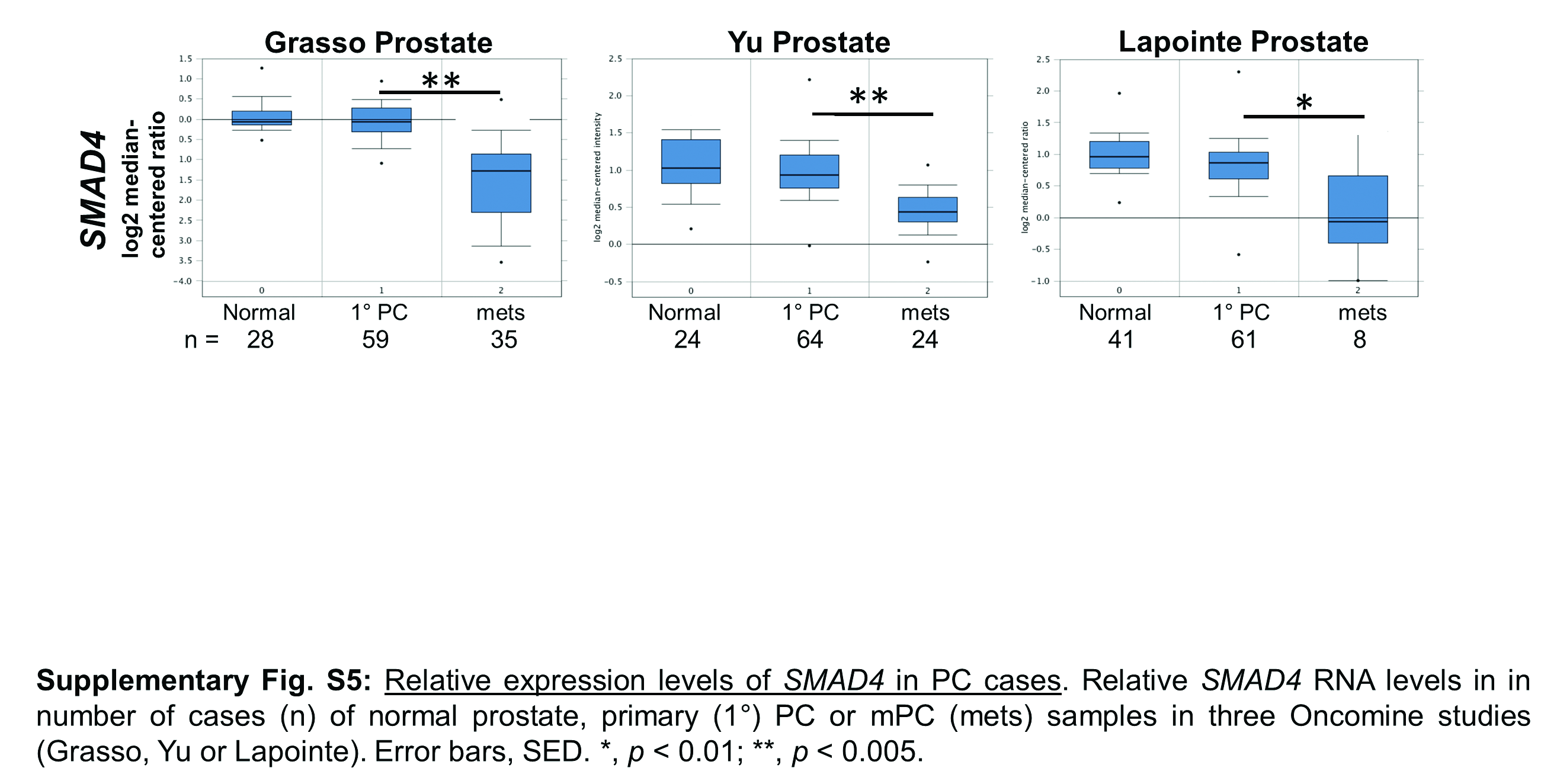

Supplement: Supplementary file 5 — Supplementary Fig. S5 [file 41388_2023_2875_MOESM5_ESM.tif]
