# Supplementary material for: PTEN-regulated PI3K-p110 and AKT isoform plasticity controls metastatic prostate cancer progression
Source: Oncogene. 2023 Oct 24;43(1):22–34. doi: 10.1038/s41388-023-02875-4 (PMC10766561; doi:10.1038/s41388-023-02875-4)
Supplement: Supplementary file 7 — Supplementary Table S2 [file 41388_2023_2875_MOESM7_ESM.pdf]

Supplementary Table S2: siRNA sequences and qPCR primers used in this study

Gene Target	Sequence[a]	Source
AKT1 (human, mouse)	S: 5’-CGCGUGACCAUGAACGAGUUU[dT][dT]-3’<br>AS: 5’-AAACUCGUUCAUGGUCACGCG[dT][dT]-3’	Sigma-Aldrich
AKT2 (human)	S: 5’-GACCCAACACCUUUGUCAUAC[dT][dT]-3’<br>AS: 5’-GUAUGACAAAGGUGUUGGGUC[dT][dT]-3’	Sigma-Aldrich
Akt2 (mouse) #2	S: 5’-GGGCCAAAGUGACCAUGAAUGACUU[dT][dT]-3’<br>AS: 5’-AAGUCAUUCAUGGUCACUUUGGCCC[dT][dT]-3’	Zhang *et al*. (84)
Akt2 (mouse) #3	S: 5’-CCACUGGCCGCUAUUAUGCCAUGAA[dT][dT]-3’<br>AS: 5’-UUCAUGGCAUAAUAGCGGCCAGUGG[dT][dT]-3’	Zhang *et al*. (84)
AKT3 (human)	S: 5’-CCAAAGCCAAACACAUUUAUA[dT][dT]-3’<br>AS: 5’-UAUAAAUGUGUUUGGCUUUGG[dT][dT]-3’	Sigma-Aldrich
SMAD4 (human, mouse) #96	S: 5’-ACUGCUAAAUUCUAUGUUAAA[dT][dT]-3’<br>AS: 5’-UUUAACAUAGAAUUUAGCAGU[dT][dT]-3’	pGIPZ shRNA V2LHS_37196[b]
SMAD4 (human, mouse) #95	S: 5’-GCGGUCUUUGUACAGAGUUAC[dT][dT]-3’<br>AS: 5’-GUAACUCUGAUCAAAGACCGC[dT][dT]-3’	pGIPZ shRNA V2LHS_37195
Smad4 (mouse) #28	S: 5’-GCAGACAGAAACUGGAUUAAACUC[dT][dT]-3’<br>AS: 5’-GAGUUUAAUCCAGUUUCUGUCUGC[dT][dT]-3’	Ding *et al*. (47)
Smad4 (mouse) #29	S: 5’-CCUGAGUAUUGGUGUUCCAUUCUC[dT][dT]-3’<br>AS: 5’-GAGAAUGGAACACCAAUACUCAGG[dT][dT]-3’	Ding *et al*. (47)
Negative Control	S: 5’-AGUGUCCUUGCUGAACGAC[dT][dT]-3’<br>AS: 5’-GUCGUUCAGCAAGGACACU[dT][dT]-3’	Sigma-Aldrich
b-actin (mouse)	F: 5'-CAGCTGAGAGGGAAATCGTG-3'<br>R: 5'-CGTTGCCAATAGTGATGACC-3'	Havens et al. (51)
Alu (human)	F: 5'-CATGGTGAAACCCCGTCTCTA-3'<br>R: 5'-GCCTCAGCCTCCCGAGTAG-3'	Havens et al. (51)

[a], S- sense strand; AS- anti-sense strand; F- forward; R- reverse

[b], siRNA sequence derived from Dharmacon shRNA clone.
